# Radiculopatia cervical de nível único com ou sem artrodese: uma revisão sistemática e metanálise

**DOI:** 10.1055/s-0046-1820486

**Published:** 2026-07-28

**Authors:** Maria Florencia Deslivia, Sherly Desnita Savio, Benedictus Satmoko, I Gusti Lanang Ngurah Agung Artha Wiguna, Ifran Saleh

**Affiliations:** 1 Centro de Osso e Articulação, St Carolus Hospital, Jacarta, Indonésia; 2 Departamento de Ortopedia e Traumatologia, Faculdade de Medicina, Udayana University, Prof. I. G. N. G. Ngoerah Hospital, Bali, Indonésia

**Keywords:** artrodese, descompressão cirúrgica, foraminotomia, metanálise, radiculopatia, vértebras cervicais, cervical vertebrae, decompression, surgical, foraminotomy, meta-analysis, radiculopathy, spinal fusion

## Abstract

**Objetivo:**

Comparar objetivamente a eficácia da descompressão para tratamento da radiculopatia cervical de nível único com e sem artrodese, com foco em estudos clínicos randomizados.

**Métodos:**

Esta análise incluiu seis estudos randomizados controlados. Uma revisão sistemática e metanálise foi realizada seguindo as diretrizes Preferred Reporting Items for Systematic Reviews and Meta-Analyses (PRISMA). O risco de viés foi avaliado, os dados foram organizados em tabelas predefinidas e os cálculos foram feitos utilizando o *software* Review Manager (RevMan).

**Resultados:**

A análise de 726 pacientes (artrodese sim: 374 vs. não: 352) não evidenciou diferenças significativas nas taxas de cifose ( *p* = 0,11), satisfação ( *p* = 0,73), complicações ( *p* = 0,53), recidiva ( *p* = 0,63) ou reoperação ( *p* = 0,36). O alívio da dor após a cirurgia e os desfechos funcionais foram semelhantes entre as técnicas.

**Conclusões:**

As técnicas com e sem artrodese apresentaram eficácia comparável para tratamento da radiculopatia cervical de nível único. Entretanto, as características específicas de cada paciente devem ser consideradas. Nível de Evidência: I.

## Abstract (en)

**Objective:**

To objectively compare the efficacy of single-level cervical radiculopathy decompression with and without fusion, with a focus on randomized controlled trials.

**Methods:**

There were six randomized-controlled studies included in the analysis. A systematic review and meta-analysis were conducted following the Preferred Reporting Items for Systematic Reviews and Meta-Analyses (PRISMA) guidelines. The risk of bias was assessed, data were organized into predefined tables, and calculations were performed using the Review Manager (RevMan) software.

**Results:**

From the analysis of 726 patients (Fusion: 374, Non-fusion: 352), no significant differences were found in kyphotic rate ( *p* = 0.11), satisfaction rate ( *p* = 0.73), complication rate ( *p* = 0.53), recurrence ( *p* = 0.63), or reoperation rate ( *p* = 0.36). Postoperative pain relief and functional outcomes were comparable between techniques.

**Conclusions:**

Fusion and nonfusion techniques offer comparable efficacy for single-level cervical radiculopathy, however patient-specific considerations are needed. Level of Evidence I.

## Pontos-Chave

- Abordagens sem artrodese demonstraram melhor relação custo-benefício em comparação a técnicas de artrodese para tratamento da radiculopatia cervical; entretanto, ainda não há consenso quanto ao método mais eficaz para essa doença.
- A técnica sem artrodese apresentou taxas de cifose, satisfação, complicações, recidiva e reoperação, assim como alívio da dor após a cirurgia e desfechos funcionais, comparáveis aos métodos com artrodese.
- A continuidade das pesquisas é essencial para aprimorar essas técnicas e ampliar a base de evidências, contribuindo para a tomada de decisão clínica informada.

## Introdução

A radiculopatia cervical, doença caracterizada por compressão da raiz nervosa na coluna cervical, frequentemente provoca dor significativa, déficits neurológicos e diminuição da qualidade de vida. 1 De modo geral, a intervenção cirúrgica é indicada após o insucesso do tratamento conservador, e seu principal objetivo é o alívio da compressão radicular e a recuperação funcional. Dentre as opções cirúrgicas, a discectomia e artrodese cervical anterior (DACA) é bastante aceita, sendo reconhecida por sua capacidade de estabilização da coluna após a descompressão. 2 3 4

Embora a DACA tenha demonstrado desfechos favoráveis em diversos estudos, há preocupações quanto às complicações associadas à artrodese e ao possível aumento da degeneração do segmento adjacente (DSA), ao desenvolvimento de pseudoartrose, à instrumentação e à abordagem anterior. 5 Esses fatores têm levado a um interesse crescente em técnicas que proporcionam a preservação do movimento, bem como em abordagens sem artrodese, como a discectomia cervical anterior (DCA), 6 foraminotomia cervical posterior (FCP )7 e descompressão endoscópica posterior.

As abordagens sem artrodese demonstraram melhor relação custo-benefício, principalmente devido à redução de custos diretos (instrumentais cirúrgicos e internação hospitalar) e indiretos. 8 Embora estudos prévios tenham destacado a eficácia comparativa dessas técnicas, 9 10 11 ainda não há consenso quanto ao melhor tratamento para essa doença. Esta revisão sistemática e metanálise tem como objetivo fornecer uma avaliação abrangente da literatura atual sobre a descompressão para tratamento da radiculopatia cervical de nível único, com foco em estudos clínicos randomizados. Ao comparar objetivamente desfechos clínicos e complicações, esta revisão busca subsidiar a tomada de decisão clínica e orientar pesquisas futuras em cirurgia da coluna.

## Materiais e Métodos

**Fig. 1:**
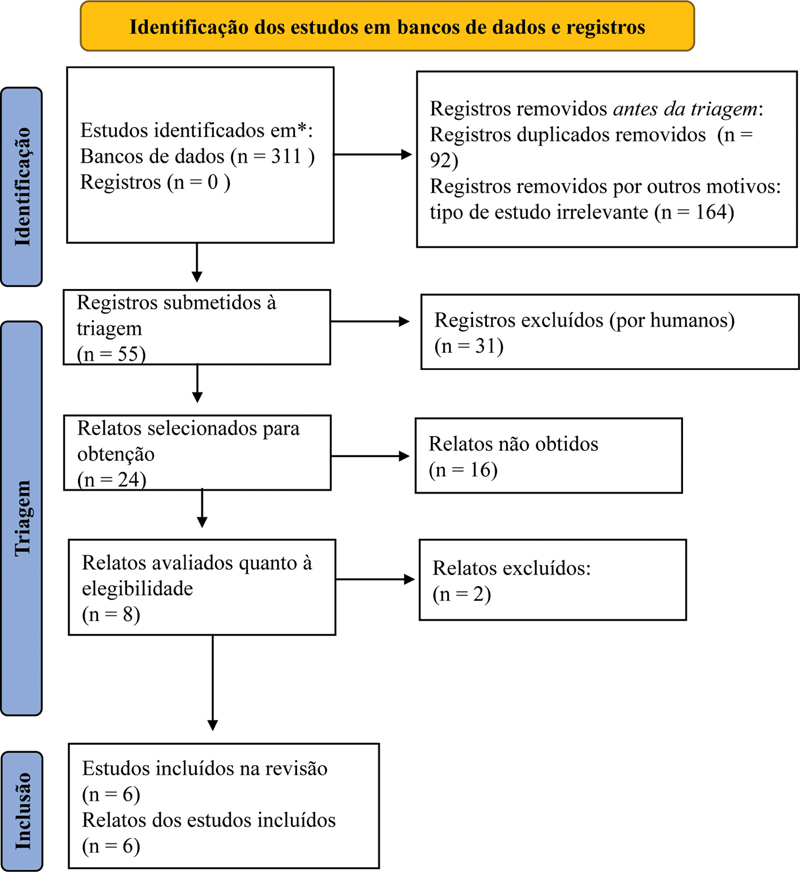
Identificação dos estudos na busca primária na literatura e fluxograma do processo de seleção.

Esta revisão sistemática e metanálise foram conduzidas em conformidade com as diretrizes Preferred Reporting Items for Systematic reviews and Meta-Analyses (PRISMA), como apresentado na Fig. 1 , além da lista de verificação e do fluxograma Quality of Reporting of Meta-analyses (QUOROM) para metanálises de estudos clínicos randomizados (ECRs). O protocolo da revisão foi elaborado e registrado no banco de dados International Prospective Register of Systematic Reviews (PROSPERO), sob o número CRD42024496863.

### Estratégia de busca e critérios de seleção

Uma busca sistemática na literatura em língua inglesa foi realizada nas bases Google Scholar, PubMed/MEDLINE, Cochrane Central Register of Controlled Trials (CENTRAL) e ClinicalTrials.gov. A última busca foi conduzida em 15 de janeiro de 2025. A estratégia de busca combinou descritores Medical Subject Headings (MeSH) e palavras-chave livres relacionadas à radiculopatia cervical e às técnicas cirúrgicas, incluindo *cervical radiculopathy* (“radiculopatia cervical”), *anterior cervical discectomy and fusion* (“discectomia e artrodese cervical anterior”) e *randomized controlled trial* (“ensaio clínico randomizado”), com uso dos operadores booleanos AND/OR (E/OU) conforme apropriado.

Os filtros aplicados incluíram estudos publicados em língua inglesa, realizados em seres humanos e classificados como clínicos randomizados. Não houve restrição quanto ao ano de publicação e apenas artigos disponíveis como texto completo foram considerados elegíveis. Os artigos foram selecionados com base nos critérios de inclusão e exclusão previamente estabelecidos, de acordo com o método População, Intervenção, Comparação e Desfecho, do inglês “Outcome” (PICO), como mostrado na Tabela 1 .

**Tabela 1:** Critérios de inclusão e exclusão segundo o método PICO

	Critérios de inclusão	Critérios de exclusão
**População**	Adultos com radiculopatia cervical de nível único após descompressão cirúrgica	Estudos com animais; Lesão traumática; Reoperações; Doença congênita ou neoplásica subjacente.
**Intervenção**	Técnicas de descompressão sem artrodese (discectomia cervical anterior, foraminotomia posterior, descompressão endoscópica).	Procedimentos não cirúrgicos; Tratamento farmacológico e nutricional; Fisioterapia ou reabilitação isolada.
**Controle**	DACA	
**Desfecho**	Desfecho clínico: satisfação, complicação, recidiva, dor, escore funcional; Desfecho radiológico: deformidade cifótica.	Estudo em andamento, sem relato de resultados; Medidas de desfecho não relatadas por completo.
**Publicação**	Estudos em inglês em publicações revistas por pares.	Resumos, editoriais, cartas; Publicações duplicadas do mesmo estudo, sem relato de desfechos diferentes; Trabalhos apresentados em congressos ou conferências.
**Delineamento experimental**	Estudos controlados randomizados.	Revisão de literatura; Estudos não comparativos.

**Abreviações:** DACA, discectomia e artrodese cervical anterior; PICO, População, Intervenção, Controle, Desfecho ( *Outcome* ).

### Seleção dos estudos e extração de dados

A seleção dos estudos foi realizada de forma independente por dois revisores. Os títulos e resumos identificados na busca eletrônica foram avaliados quanto à elegibilidade e, a seguir, pela análise do texto completo dos artigos com possível relevância. A extração dos dados também foi conduzida de forma independente pelos mesmos dois revisores, utilizando um formulário padronizado. Eventuais discordâncias em qualquer etapa do processo foram resolvidas por consenso; quando não alcançado, um terceiro revisor foi consultado.

As seguintes variáveis foram extraídas sistematicamente de cada estudo incluído: características do estudo (primeiro autor, ano de publicação, delineamento experimental), dados demográficos dos pacientes (tamanho da amostra, idade, sexo), características cirúrgicas (nível cervical tratado, tipo de técnica cirúrgica), estado neurológico pré-operatório, tempo de acompanhamento, desfechos radiológicos, desfechos clínicos, complicações pós-operatórias, taxa de recidiva e taxa de reoperação.

### Avaliação da qualidade

Os ECRs incluídos foram avaliados quanto à qualidade por dois revisores independentes, com base nos 13 itens do 2015 Updated Method Guideline for Systematic Reviews do Cochrane Back and Neck Group. 12

O risco de viés foi avaliado para determinar a elegibilidade dos estudos, utilizando as ferramentas Risk of Bias in Nonrandomized Studies – Interventions (ROBINS-I) e Risk of Bias 2.0 (RoB 2.0), da Cochrane. Os domínios avaliados foram processo de randomização, desvios das intervenções propostas, ausência de dados de desfecho, mensuração dos desfechos e seleção dos resultados reportados.

### Síntese dos dados

Os dados relativos às características dos pacientes e dos estudos (por exemplo, idade, sexo, nível tratado e déficit neurológico pré-operatório), bem como aos desfechos avaliados, de cada publicação selecionada foram extraídos e agregados. As variáveis dicotômicas foram analisadas por meio do cálculo da razão de chances (RC) e seus respectivos intervalos de confiança (IC) de 95%. Os cálculos foram realizados utilizando o software Review Manager (RevMan, Nordic Cochrane Centre, Cochrane Collaboration), versão 5.3. O modelo de efeito fixo foi adotado quando a heterogeneidade (I ^2^ ) foi < 50%, e o modelo de efeitos aleatórios quando I ^2^ > 50%.

## Resultados

### Busca na literatura e características dos estudos

A primeira busca eletrônica identificou 311 registros. Após a remoção de duplicatas e a triagem de títulos e resumos, 8 estudos foram submetidos à avaliação do texto completo. Um estudo 13 foi excluído por baixa qualidade metodológica e outro 14 por relatar desfechos provenientes da mesma coorte de pacientes de um estudo já incluído, porém com maior tempo de acompanhamento. 15 Assim, a análise final foi concentrada em 6 estudos.

### Características basais

Este estudo analisou 726 pacientes, com mais pacientes no grupo submetido à artrodese (sim: 374 vs. não: 352). A amostra foi composta predominantemente por homens, com idade entre 28 e 67 anos. O nível C6-C7 foi o tratado com maior frequência, seguido por C5-C6. Em todos os estudos, mais da metade dos pacientes apresentava déficit sensitivo no período pré-operatório, enquanto 25 a 75% apresentavam déficit motor. O tempo de acompanhamento variou de 24 a 69 meses ( Tabela 2 ).

**Tabela 2:** Características basais das amostras dos estudos incluídos

N	Autor (ano)	Intervenção	Tamanho da amostra (n)		Sexo (M:F)		Idade média (anos)		Nível tratado			Déficit neurológico	Acompanhamento (meses)
			I	C	I	C	I	C	I	C	I	C	
**1**	Savolainen et al. (1998) 16	Discectomia sem artrodese	31	DACA: 30 DACAI: 30	20:11 (M: 64,5%)	DACA: 22:8 (M: 73%) DACAI: 21:9 (M: 70%)	46	DACA: 47,9 DACAI: 49,7	C6-7 mais comum (61,3%), seguido por C5-6 (32,26%)	C6-7 mais comum (46,67%), seguido por C5-6 (38,33%)	100% déficit sensorial, 67,74% déficit motor	78,3% déficit sensorial, 75% déficit motor	36–60 (média: 48)
**2**	Wirth et al. (2000)	FCP DCA sem artrodese	47	25	26:21 (M: 55,3%)	14 (M: 56%)	30–67	28–63	C6-7 mais comum (48,9%), seguido por C5-6 (34%)	C5-6 mais comum (48%), seguido por C6-7 (40%)	78,7% déficit sensorial, 42,55% déficit motor	84% déficit sensorial, 44% déficit motor	53–69
**3**	Xie et al. (2007)	Discectomia sem artrodese	12	DACA: 15 DACAI: 15	5:7 (M: 41,67%)	DACA: 9:6 (M: 60%) DACAI: 14:1 (M: 93,3%)	42 ± 8	DACA: 42 ± 8 DACAI: 43 ± 8	C6-7 mais comum (58,3%), seguido por C5-6 (33,3%)	C5-6 mais comum (56,67%), seguido por C5-6 (36,67%)	58,3% déficit sensorial, 25% déficit motor	80% déficit sensorial, 36,67% déficit motor	24
**4**	Ruetten et al. (2008) 17	FCP totalmente endoscópica	89	86	Inicialmente, 68 (34% homens) dentre 200 pacientes		43 (27-62)		C6-7 mais comum (27,5%), seguido por C5-6 (10%)		C6-7 mais comum (30,5%), seguido por C5-6 (11%)	NR	24
**5**	Ruetten et al. (2009) 18	DACAE	54	49	Inicialmente, 43 (35,8% homens) dentre 120 pacientes		30–61		C5-6 mais comum (53,7%), seguido por C6-7 (37%)		C5-6 mais comum (53%), seguido por C6-7 (42,8%)	NR	24
**6**	Broekema et al. (2023) 15	FCP	119	124	53:66 (45% homens)	66:58 (53% homens)	51,6 ± 8,5	51 ± 8,3	C6-7 mais comum (98%)	C7 mais comum (50%), seguido por C6 (49%)	66% déficit sensorial, 36% déficit motor	66% déficit sensorial, 41% déficit motor	24

**Abreviaturas:** C, controle; DACA, discectomia e artrodese cervical anterior; DACAE, discectomia e artrodese cervical anterior totalmente endoscópica; DACAI, Discectomia e artrodese cervical anterior com instrumentação; FCP, foraminotomia cervical posterior; I, intervenção; F, sexo feminino; M, sexo masculino; NR, não relatado.

### Desfecho radiológico

**Fig. 2:**
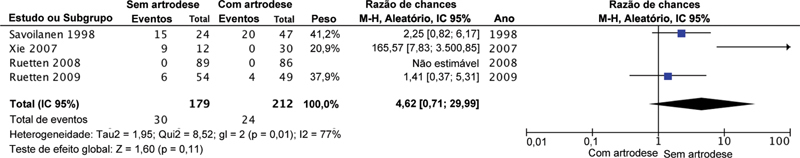
Gráfico de floresta dos desfechos radiológicos (taxa de cifose). **Abreviações:** Aleatório, modelo de efeitos aleatórios; gl, graus de liberdade; IC, intervalo de confiança; M-H, Mantel-Haenszel.

As Tabelas 3.1 e 3.2 apresentam a comparação dos desfechos entre os estudos incluídos. O desfecho radiológico foi avaliado por meio da taxa de cifose; não houve diferença estatisticamente significativa entre as técnicas com e sem artrodese para o tratamento da radiculopatia cervical (diferença média [DM] = 4,62; IC95% = 0,71–29,99; I ^2^ = 77%; *p* = 0,11), como apresentado na Fig. 2 .

**Tabela 3.1:** Análise de desfecho dos estudos incluídos

N	Autor (ano)	Desfecho radiológico (taxa de cifose)		Taxa de satisfaças		Taxa de complicações		Complicações mais comuns
		I	C	I	C	I	C	
**1**	Savolainen et al. (1998) 16	15/24 (62,5%)	20/47 (42,55%)	23/30 (76%)	45/58 (77,6%)	1/31 (3,2%)	56/60 (93,3%)	Dor grave na crista ilíaca no grupo controle (80%)
**2**	Wirth et al. (2000)	NR		NR		NR		NR
**3**	Xie et al. (2007)	9/12 (75%)	0 (0%)	NR		NR		NR
**4**	Ruetten, et al, (2008) 17	0 (0%)	0 (0%)	86/89 (96%)	78/86 (91%)	Nenhuma complicação grave, complicações moderadas em 3/89 (3,3%)	Nenhuma complicação grave, complicações moderadas em 5/86 (5,8%)	Dificuldade transitória de deglutição no grupo controle (3,5%), hipoestesia transitória relacionada a dermátomo no grupo de intervenção (3%); nenhuma complicação grave.
**5**	Ruetten et al. (2009) 18	6 (11,8%)	4 (8,3%)	49 (90,7%)	43 (87,8%)	2 (3,7%)	7 (14,3%)	Dificuldade transitória de deglutição em 6,8% (mais comum no grupo controle), hematoma superficial em 4% do grupo controle; nenhuma complicação grave.
**6**	Broekema et al. (2023) 5	NR		70 (73%)	76 (77%)	36 (30%)	35 (28%)	Sintomas no ombro (5,8%), dor radicular persistente no braço sem necessidade de cirurgia (4,1%), disfagia (2,9%) e infecção da ferida (2,9%)

**Abreviaturas:** C, controle; I, intervenção; NA, não relatada(s).

**Tabela 3.2:** Análise de desfecho dos estudos incluídos

N	Autor (ano)	Taxa de recidiva		Taxa de reoperação		Dor pós-operatória		NDI		EQ-5D	
		I	C	I	C	I	C	I	C	I	C
**1**	Savolainen et al. (1998) 16	1/31 (3,2%)	1/60 (1,7%)	2/31 (6,5%)	0 (0%)	NR		NR		NR	
**2**	Wirth et al. (2000)	12/47 (25,53%)	8/25 (32%)	9/47 (19,15%)	7/25 (28%)	96% com alívio completo	100% com alívio completo	NR		NR	
**3**	Xie et al. (2007)	1/12 (8,3%)	4/30 (13,3%)	NR		Braço: 1/12 (8%) Pescoço: 2 (17%)	Braço: 1/30 (3,3%) Pescoço: 7/30 (23,3%)	NR		NR	
**4**	Ruetten et al. (2008) 17	3/89 (3,4%)	0 (0%)	6/89 (6,7%)	4/86 (4,7%)	EVA média Braço: 7 Pescoço: 16	EVA média Braço: 8 Pescoço: 17	NR		NR	
**5**	Ruetten et al. (2009) 18	2 (3,7%)	0 (0%)	4 (7,4%)	2 (6,1%)	EVA média Braço: 8 Pescoço: 15	EVA média Braço: 10 Pescoço: 14	NR		NR	
**6**	Broekema et al. (2022) 15	NR	6 (5%)	4 (3%)	EVA média Braço: 18,6 ± 22,9 Pescoço: 24,4 ± 27,5	EVA média Braço: 15,8 ± 23,7 Pescoço: 21,7 ± 26,1	17,6 ± 14,6	19,2 ± 16,5	0,84 ± 0,15	0,82 ± 0,14

**Abreviações:** C, controle; EQ-5D, EuroQol five-dimensional questionnaire; EVA, escala visual analógica; I, intervenção; NA, não relatado(a)(s); NDI; Neck Disability Index.

### Desfechos clínicos e satisfação do paciente

**Fig. 3:**
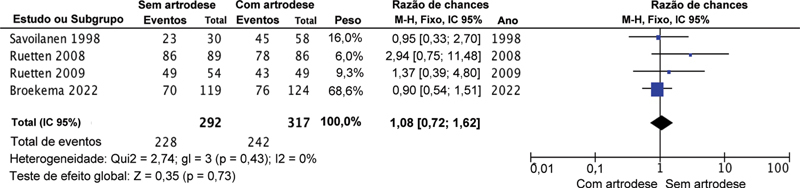
Gráfico de floresta da taxa de satisfação. **Abreviações:** Fixo, modelo de efeitos fixos; gl, graus de liberdade; IC, intervalo de confiança; M-H, Mantel-Haenszel.

A taxa de satisfação foi semelhante entre as duas técnicas (DM: 1,08; IC95%: 0,72–1,62; I ^2^ = 0%; *p* = 0,73), como apresentado na Fig. 3 .

### Complicações

**Fig. 4:**
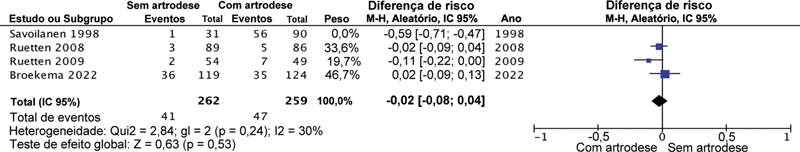
Gráfico de floresta da taxa de complicações. **Abreviações:** Fixo, modelo de efeitos fixos; gl, graus de liberdade; IC, intervalo de confiança; M-H, Mantel-Haenszel.

Os estudos incluídos relataram as complicações pós-operatórias. No grupo artrodese, um estudo descreveu a morbidade relacionada ao sítio doador do enxerto da crista ilíaca. 16 A primeira análise combinada demonstrou heterogeneidade elevada (I ^2^ = 96%). Após a exclusão desse estudo, observou-se uma redução (I ^2^ = 30%), sem diferença estatisticamente significativa nas taxas de complicações entre as técnicas com e sem artrodese (DM: -0,02; IC95%: -0,08 a 0,04; *p* = 0,53), como apresentado na Fig. 4 . Outras complicações relatadas incluíram disfagia transitória, hematoma, dor radicular em membro superior e infecção de ferida operatória.

### Taxas de recidiva e reoperação

**Fig. 5:**
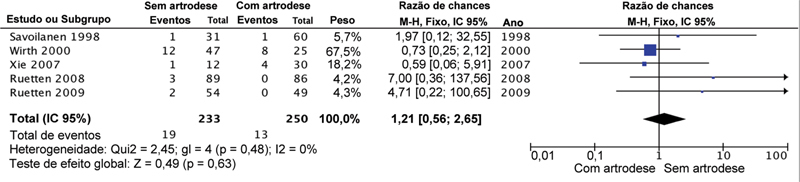
Gráfico de floresta da taxa de recidiva. **Abreviações:** Fixo, modelo de efeitos fixos; gl, graus de liberdade; IC, intervalo de confiança; M-H, Mantel-Haenszel.

Não foram observadas diferenças estatisticamente significativas entre as técnicas com e sem artrodese quanto à taxa de recidiva ( *p* = 0,63) ou de reoperação ( *p* = 0,36), como apresentado na Fig. 5 .

### Desfechos funcionais

Um estudo relatou os desfechos funcionais avaliados por meio do Neck Disability Index (NDI) e do EuroQol five-dimensional questionnaire (EQ-5D). Não foram identificadas diferenças estatisticamente significativas entre as técnicas com e sem artrodese em relação a esses desfechos.

## Discussão

Este estudo é o primeiro a sintetizar evidências de alto nível sobre os avanços recentes no tratamento da radiculopatia cervical de nível único. A análise de seis ECRs demonstrou que as técnicas sem artrodese—incluindo discectomia, FCP e DCA—geram desfechos comparáveis às técnicas com artrodese no que se refere às taxas de cifose, satisfação dos pacientes, complicações, recidiva e reoperações. No entanto, a maior exploração desses achados é necessária para maior contextualização e aprofundamento clínico.

### Desfecho clínico

A DACA é considerada o “padrão-ouro” para o tratamento da radiculopatia cervical por ter desfechos clínicos bem documentados, variando de bons a excelentes. Entretanto, estudos conduzidos por Ruetten et al. 17 18 indicam que abordagens sem artrodese, como a FCP, alcançam melhoras significativas e contínuas semelhantes. Vale notar que o tempo de internação foi menor nas técnicas sem artrodese (3 dias sem artrodese vs. 7 com), possivelmente devido ao caráter menos invasivo dessas abordagens e ao consequente menor dano aos tecidos moles.

Diversos estudos não randomizados relataram que a FCP apresenta desfechos comparáveis ou até superiores aos da DACA. 19 20 No entanto, a ausência de randomização pode introduzir um viés de seleção, uma vez que a FCP pode ter sido preferencialmente indicada para pacientes com doença mais favorável, como hérnias discais moles e laterais ou altura discal preservada, que são, por natureza, mais responsivas à descompressão isolada. Outro estudo comparando a discectomia cervical endoscópica percutânea (DCEP) e a DACA relatou taxas de revisão ligeiramente mais elevadas no grupo DCEP. 21 Vale destacar que os procedimentos de DCEP foram realizados por cirurgiões endoscopistas experientes, enquanto os casos de DACA foram conduzidos por um grupo mais amplo, com diferentes níveis de experiência. Esses fatores técnicos e metodológicos podem introduzir viés em favor das técnicas sem artrodese e limitar a generalização dos achados.

Para minimizar o viés inerente a delineamentos não randomizados, esta metanálise concentrou-se principalmente em ECRs. Contudo, apesar da robustez desse delineamento experimental, ainda há limitações metodológicas que podem introduzir viés em favor das abordagens sem artrodese. Em primeiro lugar, não houve cegamento de pacientes ou cirurgiões quanto à intervenção a ser realizada, o que aumenta o risco de viés de desempenho e detecção, particularmente em desfechos subjetivos, como satisfação e retorno ao trabalho. De modo geral, procedimentos sem artrodese são associados a incisões menores e menor alteração tecidual, o que pode reduzir as taxas de complicações e melhorar a percepção dos pacientes.

Além disso, o recrutamento dos pacientes pode ter sofrido viés de seleção. Alguns cirurgiões manifestaram preferência por um dos braços de tratamento, predominantemente pela DACA, o que pode ter influenciado as decisões de elegibilidade. 15 Segundo, o procedimento foi realizado sem placa anterior, utilizando apenas espaçadores intersomáticos ( *cages* ) de polieteretercetona (PEEK). 14 15 Embora isso reflita determinadas práticas regionais, a ausência da placa pode comprometer a estabilidade segmentar e as taxas de artrodese, talvez subestimando a eficácia da DACA. Ainda assim, metanálises anteriores 22 sugerem que os desfechos de construções utilizando somente espaçadores intersomáticos não são inferiores àquelas com placa, embora isso dependa do contexto clínico.

Os desfechos radiológicos, incluindo o alinhamento cifótico, não demonstraram diferenças clinicamente significativas entre as técnicas com ou sem artrodese. Da mesma forma, medidas funcionais, como o NDI e as escalas visuais analógicas (EVAs) para dor cervical e no membro superior, evidenciaram melhora substancial após as duas abordagens. Além dessas métricas objetivas, os desfechos relatados pelos pacientes (qualidade de vida, retorno ao trabalho e satisfação global) foram similarmente favoráveis. 19 23 Isso reforça a importância do planejamento terapêutico individualizado, baseado nas preferências do paciente e em cada quadro clínico.

### Complicações, recidivas e reoperações

Embora as taxas globais de complicações entre os grupos com e sem artrodese tenham sido semelhantes, a natureza dessas complicações foi diferente. Procedimentos com artrodese foram associados à maior frequência pós-operatória de disfagia, hematoma e paralisia transitória unilateral do nervo laríngeo recorrente. Em contrapartida, as técnicas sem artrodese apresentaram maiores taxas de hematoma superficial e complicações como ombro congelado e tendinite supraespinhal, em especial nos casos de FCP. 14 15 As reoperações no nível índice e as infecções da ferida operatória também foram mais prevalentes nos procedimentos sem artrodese, ressaltando a importância da técnica cirúrgica criteriosa e da seleção adequada dos pacientes.

Lin et al. 23 observaram que, embora a DACA e a FCP tenham proporcionado alívio significativo da dor, o grupo não submetido à artrodese apresentou maior incidência de procedimentos secundários relacionados à reestenose. MacDowall et al. 24 destacaram ainda que, apesar de ambos os grupos terem alcançado reduções significativas nos escores do NDI ao longo de 5 anos, a taxa de reoperação foi maior no grupo sem artrodese (aproximadamente 1/10) em comparação ao grupo com artrodese (cerca de 1/25). Foster et al. 25 corroboraram esses achados ao demonstrar desfechos comparáveis entre a FCP e a DCA, com diferenças mínimas nas taxas de reoperação. A dor radicular persistente no membro superior, embora sem necessidade de intervenção cirúrgica, foi mais comum nos pacientes sem artrodese. Essa observação suscita questionamentos quanto à eficácia em longo prazo das técnicas sem artrodese e destaca a necessidade de mais pesquisas para resolução de sintomas residuais.

Embora este estudo tenha se concentrado principalmente em desfechos imediatos e de curto prazo, as implicações em longo prazo, como a DSA, merecem maior investigação. Os procedimentos com artrodese estão associados ao maior risco de DSA devido à alteração da sobrecarga biomecânica, uma complicação que parece ser menos frequente nas técnicas sem artrodese. Estudos futuros devem buscar determinar se abordagens sem artrodese reduzem a incidência de DSA e as complicações relacionadas ao longo de períodos maiores de acompanhamento.

## Considerações Futuras

A relação custo-benefício das opções cirúrgicas é uma consideração cada vez mais relevante. Técnicas sem artrodese podem reduzir os custos relacionados à saúde devido ao menor tempo de internação e recuperação mais rápida. 9 12 Porém, o possível aumento das taxas de reoperação também deve ser incorporado às análises econômicas. Além disso, protocolos de reabilitação pós-operatória individualizados podem otimizar os desfechos e reduzir complicações em procedimentos com ou em artrodese, o que justifica sua investigação específica em estudos futuros.

Procedimentos minimamente invasivos inovadores têm surgido como alternativas promissoras para o tratamento da radiculopatia cervical. Estudos comparativos, incluindo os de Dunn et al. 20 e Ahn et al., 21 indicam que essas novas técnicas podem gerar desfechos clínicos equivalentes aos da DACA tradicional. Ainda assim, lacunas na base atual de evidências reforçam a necessidade de estudos mais robustos e de alta qualidade metodológica, com medidas de desfecho padronizadas e períodos de acompanhamento mais prolongados, a fim de aprimorar as estratégias cirúrgicas.

## Limitações

Esta metanálise apresenta diversas limitações. Em primeiro lugar, o número de estudos incluídos por desfecho foi limitado; além disso, que alguns desfechos clínicos relevantes (ex.: NDI, EQ-5D e cifose) foram relatados por poucos trabalhos. Consequentemente, as estimativas combinadas desses desfechos são baseadas em dados escassos e estão sujeitas a heterogeneidade considerável, o que reduz a certeza dos achados. Logo, tais conclusões devem ser interpretadas com cautela.

Segundo, o grupo sem artrodese compreendeu técnicas cirúrgicas heterogêneas, incluindo discectomia anterior sem artrodese, foraminotomia posterior e descompressão endoscópica anterior ou posterior. Embora tenham sido consideradas análises de subgrupo de acordo com técnicas específicas sem artrodese, o número limitado de estudos e a inconsistência na apresentação dos desfechos impediram comparações significativas entre subgrupos. Isso levou ao agrupamento de todos os procedimentos sem artrodese para análise. Essa heterogeneidade clínica pode ter diluído os efeitos específicos de cada técnica, contribuído para maior heterogeneidade estatística e limitado a capacidade de interpretação das estimativas de efeito combinadas, que devem ser compreendidas como um efeito médio entre diferentes procedimentos sem artrodese, e não como a comparação com uma única técnica cirúrgica.

Em terceiro lugar, em razão da natureza das intervenções cirúrgicas, o cegamento de cirurgiões e pacientes não foi factível em nenhum dos estudos incluídos, o que introduz um risco inerente de viés.

Ainda assim, os estudos apresentaram delineamento rigoroso, a maioria com avaliadores independentes dos desfechos e protocolos consistentes de acompanhamento. Ademais, estudos cirúrgicos com randomização adequada, análise por intenção de tratar e medidas objetivas de desfecho, como taxa de reoperação e perfil de complicações, continuam a dar evidências valiosas e clinicamente relevantes. Apesar das limitações inevitáveis dos ECRs cirúrgicos, esses estudos ainda apresentam o mais alto nível de evidência à disposição para orientar a tomada de decisão entre técnicas com e sem artrodese no tratamento da radiculopatia cervical de nível único.

## Conclusão

Este estudo destaca a eficácia comparável entre técnicas com e sem artrodese no tratamento da radiculopatia cervical de nível único, cada uma com vantagens e limitações distintas. Ao considerar fatores específicos do paciente, inovações cirúrgicas e desfechos em longo prazo, os clínicos podem individualizar as intervenções de forma mais precisa para obtenção dos desfechos ideais. A continuidade das pesquisas é fundamental para o aprimoramento dessas técnicas e a ampliação da base de evidências, a fim de sustentar tomadas de decisão clínicas cada vez mais fundamentadas e informadas.
